# Gender Disparity in Academic Neurosurgery

**DOI:** 10.7759/cureus.4628

**Published:** 2019-05-09

**Authors:** Tiffany Odell, Harjyot Toor, Ariel Takayanagi, Bailey Zampella, Javed Siddiqi, Sabeena Jalal, Khashayar Golbaz, Sadia Qamar, Faisal Khosa

**Affiliations:** 1 Neurosurgery, Desert Regional Medical Center, Palm Springs, USA; 2 Neurosurgery, Riverside University Health System Medical Center, Moreno Valley, USA; 3 Radiology, Vancouver General Hospital, Vancouver, CAN; 4 Radiology, University of British Columbia, Vancouver, CAN

**Keywords:** gender disparity, h-index, publications, research productivity, women in neurosurgery, women in surgery

## Abstract

Background

In the 1960s, less than 10% of medical school graduates were women. Today, almost half of all medical school graduates are women. Despite the significant rise in female medical school graduates, there continues to be a large gender gap in most subspecialties, particularly surgical subspecialties such as neurosurgery.

Objective

The purpose of our study was to assess the factors contributing to differences in the academic ranks of male and female staff in academic neurosurgery programs in Canada and the United States (US).

Methods

Data about women in academic neurosurgery was collected from a number of sources, including Fellowship and Residency Electronic Interactive Database (FREIDA), Accreditation Council for Graduate Medical Education (ACGME), Canadian Resident Matching Service (CaRMS) FRIEDA, ACGME, CaRMS, Pubmed, and Scopus, to create a database of all neurosurgeons in the US and Canada. The analysis included neurosurgeons in academic and leadership ranks and also the H index, citations, publications, citations per year, and publications per year.

Results

Women represent only 12% of neurosurgeons in the US and Canada. When gender is further analyzed by academic appointment, women represent just over 12% of neurosurgeons at the assistant and associate professor levels (15.44% and 13.27%, respectively) but significantly less at the full professor level (5.84%). Likewise, only 7.45% of women hold first-in command leadership positions while 4.69% hold second-in-command positions within their institutions.

Conclusions

The existing data shows that women are significantly under-represented in academic neurosurgery. Lack of role models, experience, limited scientific output, and aspirations of a controlled lifestyle could be the potential contributing factors.

## Introduction

In recent years, women have surpassed their male counterparts on the education forefront, earning more undergraduate degrees than men. Women now pursue graduate-level education in all fields and, reflectively, medical school enrollment has experienced an increasing number of female applicants. In the 2015-2016 academic year, 46.4% of medical school graduates were women [1]. This is a considerable change from 1965 when women accounted for 9% of the United States (US) medical school enrollees and a meager 7% of medical school graduates [2]. Despite the numbers showing that women now represent about 50% of all medical school enrollees, women are still underrepresented in the profession, particularly in subspecialties such as anesthesiology, infectious disease, oncology, and radiology [3-6]. A study published in 2017 concluded that male North American musculoskeletal radiologists significantly outnumber their female colleagues, who are also underrepresented in professorship roles and have lower odds of attaining a high h-index score, a metric that attempts to measure both the productivity and citation impact of the publications of a scientist or scholar [4]. Another study published in the same year reported significant gender disparity in leadership among neuroradiologists [5]. Almost half of all North American dermatologists are noted to be women, yet only a quarter occupy faculty positions [7]. Women are also found to be less productive than male dermatologists in research in terms of years spent in research, the number of publications, and citations [7]. Women were shown to lag behind men in research productivity even in the field of breast imaging where they dominate [8]. The only subspecialties in which women comprise a higher percentage than men were Child and Adolescent Psychiatry, Geriatric Medicine, Pediatrics, and Obstetrics and Gynecology [9]. In particular, surgical subspecialties have the most significant gender gap. In the US, a reported 14% of women choose to go into a surgical subspecialty as compared to 33% of men [10]. Of the surgical subspecialties, this is most pronounced in Orthopedic and Thoracic surgery where women account for only 5% and 6%, respectively [9]. 2015 data from the U.S. show that women make up 7.8% of neurosurgeons [9]. In this study, we examine female neurosurgeons in the United States and Canada according to their academic title, participation in leadership, and research productivity.

## Materials and methods

The database for the academic and administrative faculty members for neurosurgery programs, across the US and Canada, was created between January to May of 2017. For US programs, the official website of Fellowship and Residency Electronic Interactive Database (FREIDA) was used as the primary resource. This database provides information about all the neurosurgery residency programs accredited by the Accreditation Council for Graduate Medical Education (ACGME) enrolled with the American Medical Association (AMA). A total of 110 neurosurgery programs listed in FREIDA online were searched for faculty listing. Out of these 110 programs, faculty listing was available for only 100 programs. Ten programs were excluded from the data collection, as the required faculty information was not available on the program's official website. Canadian Resident Matching Service (CaRMS), a national, independent organization, provides discipline and university-based enlisting of residency program descriptions in Canada. The official webpage of CaRMS was utilized as the principal source for identifying the neurosurgery programs available in Canada, by discipline and university-based search criteria. Nine enlisted programs were searched for faculty listing. Final inclusion criteria were based on the availability of faculty listing on the official webpage of the both the US and Canadian neurosurgery programs. 

Faculty listings of the division of neurosurgery, from the respective websites of the selected universities, were reviewed. The faculty listings were searched for gender, academic ranking, and departmental leadership roles. The members whose gender could not be identified from the university website were further searched utilizing their Doximity (Doximity, Inc., San Francisco, California) and LinkedIn (LinkedIn Corporation, Mountain View, California) profiles. Only the members with the academic ranking of Professor, Associate Professor and Assistant Professor with MD degrees were included. Members without these academic rankings were excluded. Adjunct and retired faculty were also excluded from the final data set. Physicians were further categorized for their departmental leadership roles, including Chief, Co-Chief, Section Chief, Director, Co-Director, Section Director, Program Director, Chair, Vice-Chair, Head, and Section-Head. Elsevier's Scopus (Elsevier, New York, US), the largest abstract and citation database for peer-reviewed literature was used to collect information about the publications, h-index, citations, and duration of the research in years for each faculty member. The average number of publications and citations per active research year for each faculty member was also calculated.

Scopus was chosen because of its reliability and consistency in measuring the h-index when compared to Google Scholar (Google, Mountain View, California) and Web of Science. Scopus has 40 million publications recorded and is the most reliable tool for calculating the h-index because of its excellence at distinguishing authors. A published study shows a high degree of correlation between H-indices calculated from Google Scholar and Scopus. The h-index is defined as an author-level metric that measures both the productivity and citation impact of the publications of a scientist or scholar. This is based on the set of the scientist’s most-cited papers and the number of citations that they have received in other publications.

Once data was obtained, the analysis was carried out using Stata software (version 14.2, StataCorp LLC, College Station, Texas). Data were tested for normality using the Kolmogorov-Smirnov test and histograms. Since the distribution was skewed, median and ranges were calculated for quantitative variables. Frequency and percentages were calculated for qualitative variables. Chi-square was applied to see the difference between gender and academic ranks and gender and leadership ranks, applying a p-value of ≤0.05 as statistically significant. The Mann-Whitney U test was used to observe the difference between males and females for the h-index, citations, publications, citations/year, and publications/year. The Kruskal-Wallis test was used to see the difference between academic ranks and leadership ranks for the h-index, citations, publications, citations per year, and publications per year. A multiple linear regression analysis was applied since the h-index was the outcome of interest and gender was the main exposure.

## Results

**Figure 1:**
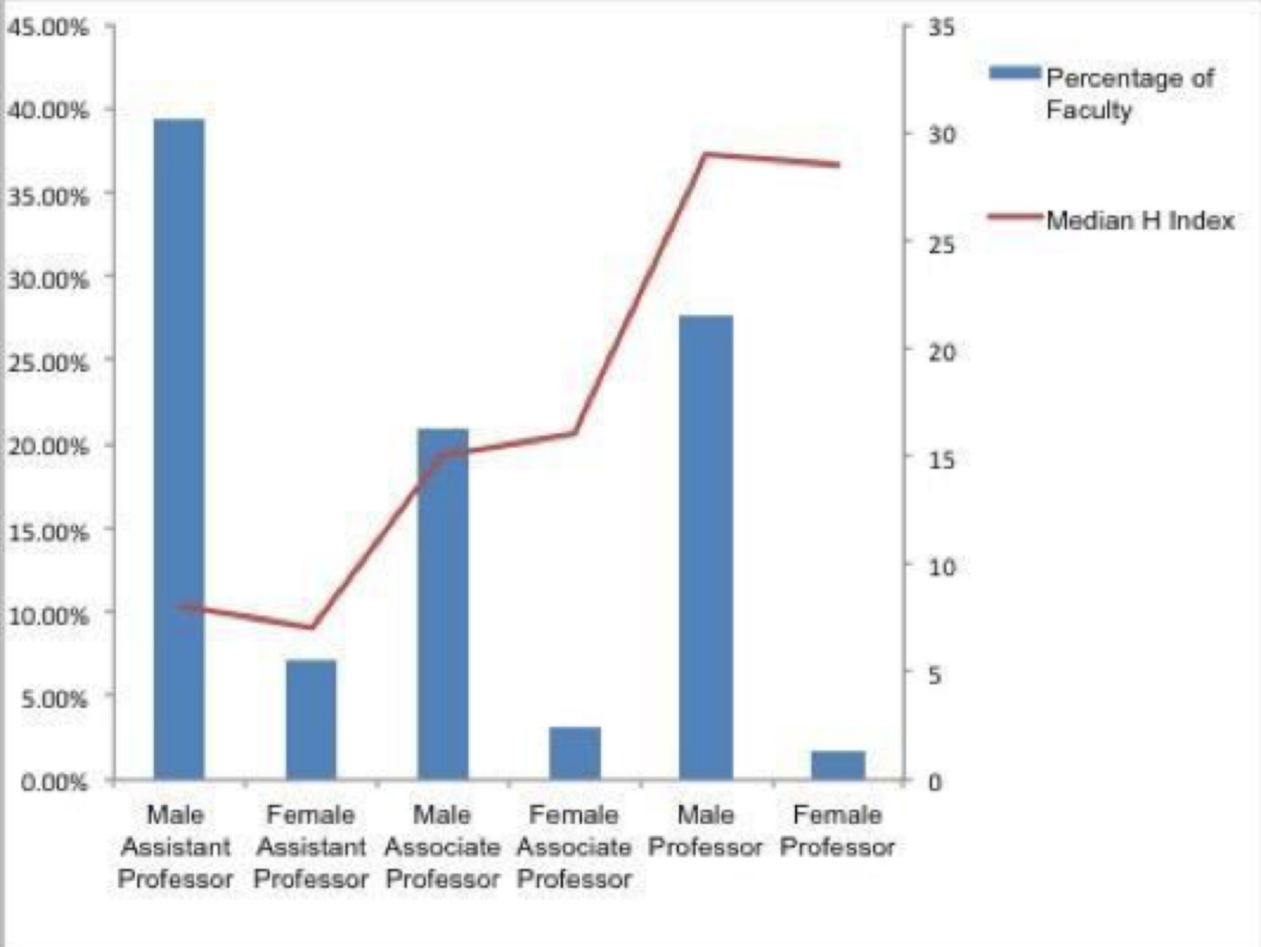
Percentage distribution of gender across leadership ranks

**Figure 2:**
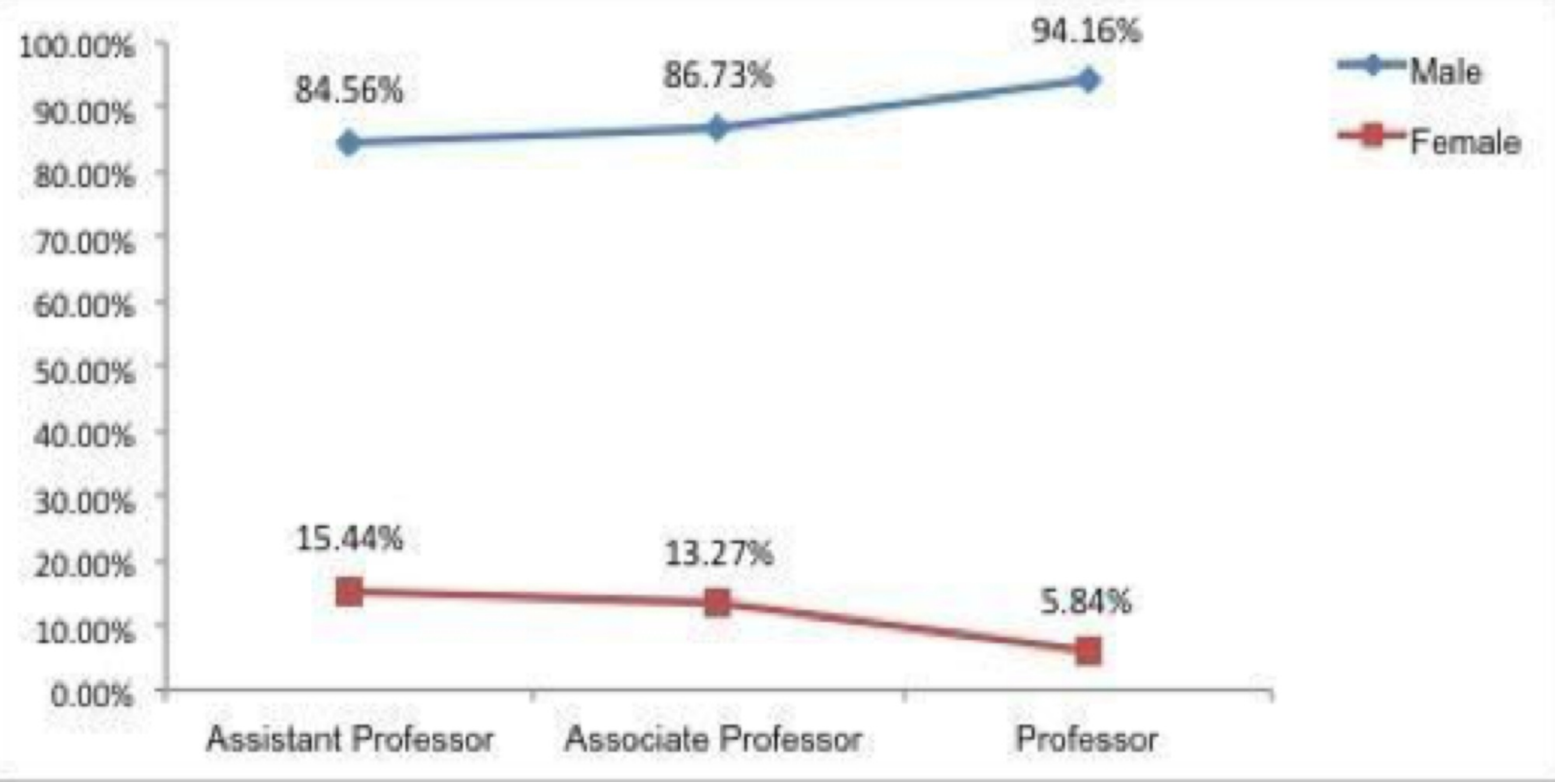
Percentage distribution of gender across academic strata

**Figure 3:**
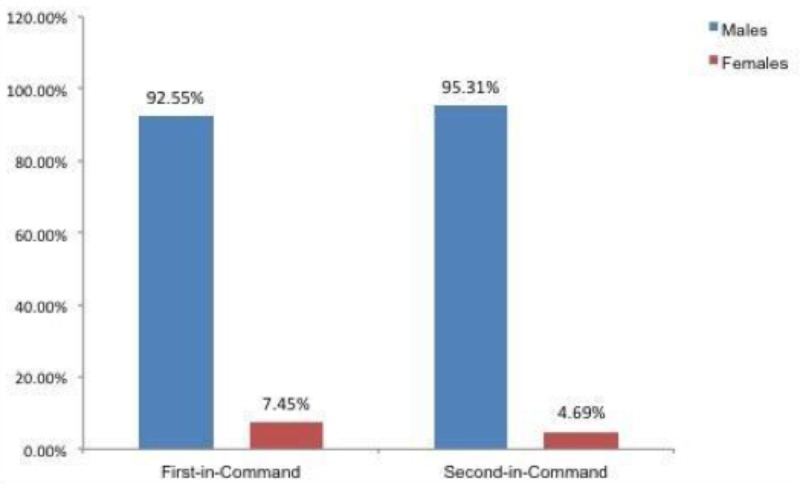
Percentage distribution of gender across academic strata

Looking at leadership ranks, we saw that 319 faculty were serving in different leadership positions. A total of 236 men (92.55%) were in first-in-command leadership positions, 19 females (7.45%) were working in first-in-command positions, 61 males (95.31%) were working in second-in-command positions, and three females (4.69%) were working in second-in-command positions, as seen in Figure 1. A total of 1811 neurosurgery faculty members in the US and Canada were identified. Of those, 1592 (87.91%) were males, and 219 (12.09%) were females. Among the 1811 neurosurgery faculty identified, 842 (46.49%) were Assistant Professors, 437 (24.13%) were Associate Professors, and 532 (29.38%) were Professors. Women held proportionately more Assistant and Associate Professorships as compared to their overall faculty male:female ratios, however, they held significantly less full Professor positions; only 5.84% as compared to 94.16% male counterparts, as noted in Figure 2. Looking at leadership ranks, we saw that 319 faculty were serving in different leadership positions; 236 men (92.55%) were in first-in-command leadership positions, 19 females (7.45%) were working in first-in-command positions, 61 males (95.31%) were working in second-in-command positions, and three females (4.69%) were working in second-in-command positions, as seen in Figure 1. In both first-in-command and second-in-command positions, men held the clear majority of postings, as seen in Figure 3.

Applying the Kruskal-Wallis test, we saw that there was a significant difference in the h-index across different academic ranks (Chi-square = 481.56, DF = 2, p-value ≤0.001). Looking at the h-index across gender, we applied the Mann-Whitney U test and saw that there was a significant difference between male and female faculty (z= 5.389, p-value ≤0.001). There was a significant difference between the total number of citations of papers of male and female faculty (z= 4.736. p-value ≤0.001). There was a significant difference in the number of publications of male and female faculty (z= 5.755, p-value ≤0.001). This significant difference was also noted even when we compared publications per year (z = 3.8, p-value ≤0.001) and citations per years (z= 2.966, p-value ≤0.001), as seen in Table 1. The overall median h-index is 15 (0-96). For all male faculty, the median h-index was 15 (0-96). For the female faculty, the median h-index was 10 (0-77).

**Table 1:** Distribution of the h-index, citations, publications, and years across gender

Variables	Academic Rank	Male (median & range)	Female (median & range)
H Index			
	Assistant Professor	8 (0 – 84)	7 (0 – 48)
	Associate Professor	15 (0 – 78)	16 (1- 47)
	Professor	29 (0 – 96)	28.5 (0 – 77)
Publications			
	Assistant Professor	21 (1 – 640)	18 (1 – 364)
	Associate Professor	47 (1 – 500)	42.5 (3 – 164)
	Professor	108.5 (1 – 1109)	79 (1 – 386)
Citations			
	Assistant Professor	290 (0 – 40022)	189 (0 – 9487)
	Associate Professor	841.5 (0 – 36235)	948.5 (1 – 7426)
	Professor	3202 (0 – 54772)	3238.5 (0 – 25763)
Years of Research			
	Assistant Professor	14 (0 – 2017)	12 (1 – 55)
	Associate Professor	20 (3 – 66)	16 (2 – 40)
	Professor	30 (2 – 66)	30 (10 – 62)
Publications per year			
	Assistant Professor	1.71 (0 – 26.38)	1.5 (0.09-22)
	Associate Professor	2.265 (0.06 – 40.73)	2.36 (0.15 – 20.5)
	Professor	3.735 (0 – 49.7)	2.67 (0.15 – 12.43)
Citations per year			
	Assistant Professor	21.94 (0 – 1143.49)	20.5 (0 – 338.82)
	Associate Professor	44.345 (0.17- 100.13)	44.22 (0.25-742.6)
	Professor	106.31 (0 – 1430.53)	89.59 (12.09 – 849.57)

The index is on the Y-axis and the regions are given on the X-axis. We can see that some provinces in Canada and some states in the US have a much higher h-index than the median cut-off value based on this data. Given in Table 2 and Table 3 are the codes that identify the names of the regions and their respective median h-index values.

**Table 2:** Median h-index distribution across state areas

Region Code	State (USA)	Median h-index	Higher than overall median h-index of 15	Median h-index for male faculty	Median h-index for female faculty	Female h-index higher than median
1	Alabama	17	√	19	10.5	
2	Arizona	7		6.5	7	
3	Arkansas	19	√	21	10	
4	California	16	√	18.5	14	
5	Colorado	12		12		
6	Columbia	12		12	10	
7	Connecticut	12.5		14.5	6	
8	Florida	16	√	16.5	11	
9	Georgia	9.5		8.5	14	
10	Illinois	24.5	√	24.5		
11	Indiana	13		14	9	
12	Iowa	13		15	11	
13	Kansas	7		8	5	
14	Kentucky	22	√	23	7	
15	Louisiana	24	√	24	29	√
16	Maryland	29	√	30.5	19	√
17	Massachusetts	8		8		
18	Michigan	18	√	19	13	
19	Minnesota	10		11	3	
20	Mississippi	11		31.5	18	√
21	Missouri	30	√	11	20	√
22	Nebraska	17	√	15	10.5	
23	New Jersey	13		19	7	
24	New Mexico	11		22	7	
25	North Carolina	21.5	√	22	8	
26	Ohio	5.5		5.5		
27	Oklahoma	15.5	√	15	20	√
28	Oregon	10		10	7	
29	Pennsylvania	10		12	6	√
30	Puerto Rico	19	√	20.5	9.5	
31	South Carolina	17	√	19	16	√
32	Tennessee	7.5		7.5	8.5	
33	Texas	20	√	22	19	√
34	Utah	22.5	√	23.5	18	√
35	Vermont	19	√	19		
36	Virginia	7		8.5	3	
37	Washington	14		15	4.5	
38	West Virginia	15		15		
39	Wisconsin	25	√	25	21	√

**Table 3:** Median h-index distribution across province areas

Region Code	Province (Canada)	Median h-index	Higher than overall median h-index of 15	Median h-index for male faculty	Median h-index for female faculty	Female h-index higher than median
1	Alberta	22.5	√	23	14	
2	British Columbia	9		7.5	13	
3	Manitoba	14		15	6.5	
4	Quebec	9		17.5	7	
5	Nova Scotia	13		30	17	√
6	Ontario	8.5		10	0	
7	Saskatchewan	6		7	5.5	

Data were tested for normality. Log transformation was done for the continuous variables of the h-index, citations, years, and number of publications, which were initially skewed in distribution. At the univariate level, simple linear regression was applied. We choose the p-value of 0.25 for the univariate level, so as to enter the maximum number of variables in the model. Each variable was regressed independently with the h-index, their assumptions were checked, and their significance was reported. Gender was our primary exposure of interest. Variables that were significant on univariate regression were gender (p-value ≤0.001), publications (p-value ≤0.001), citations (p-value ≤0.001), years of active research (p-value ≤0.001), academic ranks (p-value ≤0.001), publications per year (p-value ≤0.001), and citations per year (p-value ≤0.001). Leadership ranks (p-value = 0.55) was dropped from the model, as it was insignificant. Next, they were selected for inclusion into the multivariable linear regression analysis. We checked for multicollinearity between independent variables and were assessed using a correlation coefficient. Cramer's V test was used for one nominal and one ordinal variable, and the Spearman test was used for one continuous variable and one ordinal variable. A correlation of 0.8 was treated as the presence of multicollinearity. There was no multicollinearity seen. Main effects were identified using a stepwise selection strategy and based on the p-value, we decided to keep a variable in the model or remove it. Leadership ranks were brought forward again in the multivariable model but were again dismissed from the model (p-value = 0.19). The multivariable analysis supported the inclusion of gender, citations, publications, academic rank, and years of research in the preliminary model. The final step was to check for interaction. Interaction terms were created between each of the main effects in the model; there was significant interaction between academic ranks and publications (p-values = 0.06 and 0.08) and academic ranks and citations (p values = 0.01 and 0.71). We can deduce that the odds among the female faculty of having a higher h-index are significantly lower (OR=0.65) than the male faculty, keeping all other variables constant.

The final model revealed y (x) = β0+ β1(Gender) + β2(Publications) + β3(Citations) + β41(Academic Rank- Associate Professor) + β42(Academic Rank- Professor) + β5(Years of research) + β6 (Publications per year) + β7(Citations per year) β81 (Academic Rank Associate Professor * Publications) + β82 (Academic Rank Professor * Publications) + β91 (Academic Rank Associate Professor * Citations) + β82 (Academic Rank Professor * Citations).

This prediction equation accounted for major variability in the model as adjusted R square = 0.82, F test was 1618.1, and p-value was ≤ 0.001. The remaining variability in the model may have been explained by variables such as full-time versus part-time employment, years of employment, and contract versus tenure positions. However, this was beyond the scope of our paper, as we used the data that was available on the Internet.

## Discussion

From a historical perspective, there has been a female presence in neurosurgical-type practice dating as far back as the fifteenth century, when there are accounts of Turkish women performing solo surgical practice akin to pediatric neurosurgery [11]. In these records, women called Tabibes participated in the extraction of fetuses with hydrocephalus and macrocephaly from the womb [11]. In modern medicine, Diana Beck MD was the first female neurosurgeon. She was trained first as an apprentice under Hugh Cairns MD in 1939 at Radcliffe Infirmary in Oxford and then went on to become the first female neurosurgeon by accepting a position at Royal Free Hospital in 1943 [12]. Dr. Beck’s academic pedigree includes a degree of displacement from Harvey Cushing, who is commonly revered as the “Father of Neurosurgery” and William Halstead, who is one of the “Founding Four” professors at John’s Hopkins Hospital. In the twentieth century, women in neurosurgery have experienced exponential growth, beginning with pioneer, Ruth Kerr Jakoby MD, who is credited as being the first woman to hold an American Board of Neurological Surgery (ABNS) certification. During that same decade, 1960-1969, only one other woman joined the ranks of ABNS certification with Dr. Jakoby [12-13]. Since then, this number has grown significantly over the last 60 years, from five in the following decade (1970-1979), to 67 from 1990-1999, and now well over 200 ABNS-certified female neurosurgeons [12,14].

While the overall number of women in neurosurgery as compared to men holds steady from residency to academic appointments, we found an abrupt decrease in the number of women holding full professorships as compared to the assistant professor and associate professor titles. Among the 1,181 faculty members, men, by and large, outnumbered women (87.91% vs 12.01%, respectively). At 12%, the overall number of women in academic neurosurgery in this data set matches that of women in other studies. For example, an analysis of a cohort of medical applicants matched to neurosurgical residency between 2000-2009 found females to constitute 12% of successful applicants [15]. When we break down the data set by academic appointment, women continue to represent just over 12% of neurosurgeons at the assistant and associate professor levels, accounting for 15.44% of assistant professors and 13.27% associate professors. Interestingly, at the full professor level, there is a significant drop in the proportion of women holding these positions (only 5.84%). Likewise, there is also a significantly lower number of women holding leadership positions within their departments. Only 7.45% of women hold first-in-command positions and 4.69% hold second-in-command positions. Only two women have ever chaired academic neurosurgery departments [16]. As with many other medical subspecialties, the promotion of women to leadership positions has yet to catch up with the increase in the female population in neurosurgery as a whole. The reasons behind this have not yet been defined but are multifactorial.

There are two significant publications citing the importance of women in neurosurgery, with a call for reducing the gender bias within the specialty. One, published in 2008 by Benzil et al., examines the future of neurosurgery with a focus on the recruitment and retention of women within the field, stating that women are vital to the specialty and its growth [17]. The second, published in 2011 by Robert Spetzler MD, stresses the goal that “gender is less important than the overarching fact that we are all just Neurosurgeons" [18]. Studies such as the Benzil et al. 2008 study on the recruitment and retention of women in neurosurgery have identified several barriers hindering the entrance of women into the field of neurosurgery. These include, but may not be limited to, lifestyle concerns, scarcity of female mentorship, lack of up-to-date career programs, socio-cultural belief systems, discrimination, lack of encouragement in medical school, and early negative experiences [15,19-20]. While lifestyle is a major factor in career choice for medical students, the 80-hour week seems to have little effect on female medical students’ decision to pursue surgery as a career. The effect of the 80-hour work week specific to neurosurgery has yet to be studied [21-23].

Nonetheless, many of the issues proposed in the 2008 white paper on the recruitment and retention of women in neurosurgery still hold true. The critical mass of 15%, the percentage of female neurosurgeons, which would comprise an adequate population from which to derive female role models/mentors for aspiring young neurosurgeons, has yet to be reached. There is still a need for programs to expose medical students to female neurosurgeons, which may help to encourage female students to enter the field. Neumayer et al. noted that women who attend medical schools with higher proportions of female surgical faculty are more likely to enter surgical residency programs. To achieve this, females in leadership positions in the field of neurosurgery must also increase.

Although overall progress has been slow in recent years, there have been several major milestones. In 2016, Dawn R. Tartaglione DO became the first osteopathic neurosurgeon, and the third female, to be appointed as president of the American College of Osteopathic Surgeons. In April 2017, Shelly D. Timmons MD was the first female neurosurgeon to be named the president of the American Association of Neurological Surgeons (AANS). This represents an important symbolic change as the only major neurosurgical professional organization to name a female leader. Our study confirms the paucity of women in neurosurgery not only in overall numbers but increasingly so in higher academic positions and research productivity. Future studies could examine other potential variables such as marital status, the number of children, and the age of female neurosurgeons at various academic stages.

## Conclusions

While the overall number of women in neurosurgery as compared to men holds steady from residency to academic appointments, we found an abrupt decrease in the number of women holding full professorships as compared to the assistant professor and associate professor titles. Our study confirms the paucity of women in neurosurgery not only in overall numbers but increasingly so in higher academic positions and research productivity. This finding warrants further investigation and future studies could examine other potential variables such as marital status, the number of children, and the age of female neurosurgeons at various academic stages.
